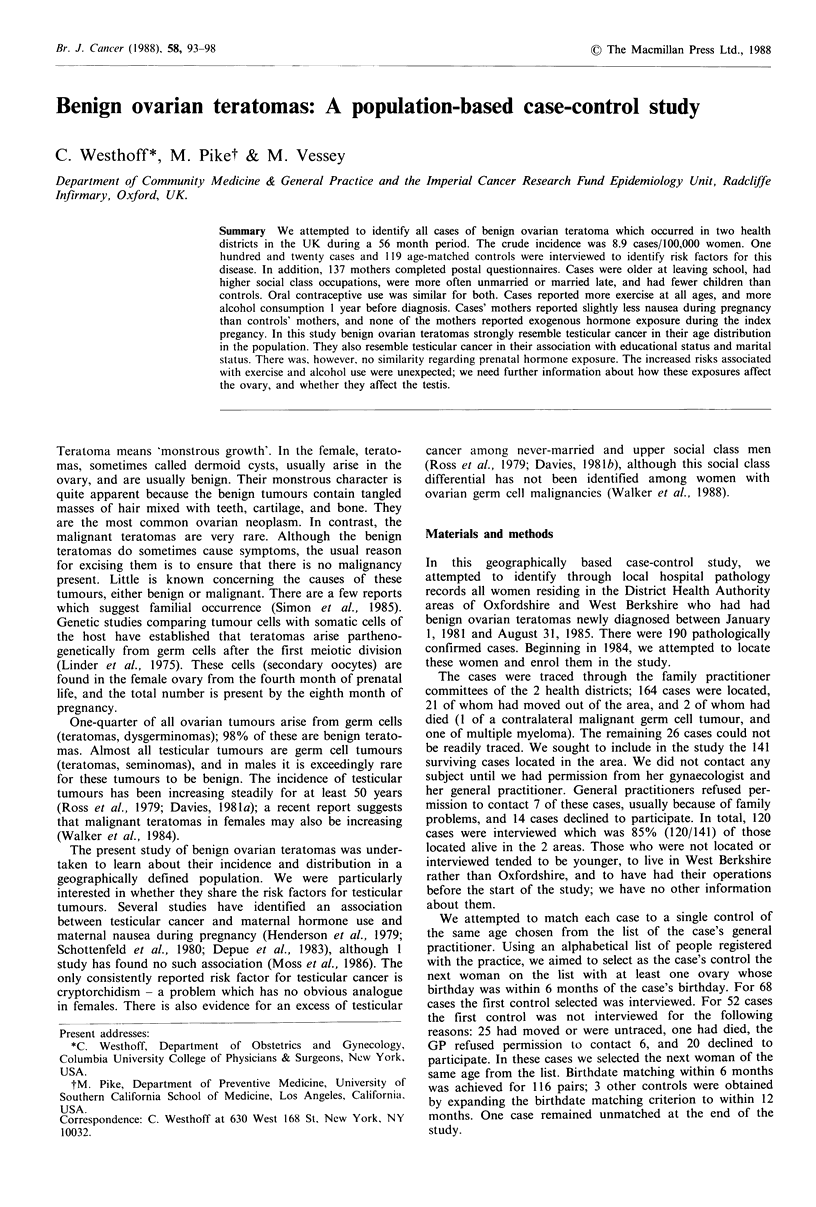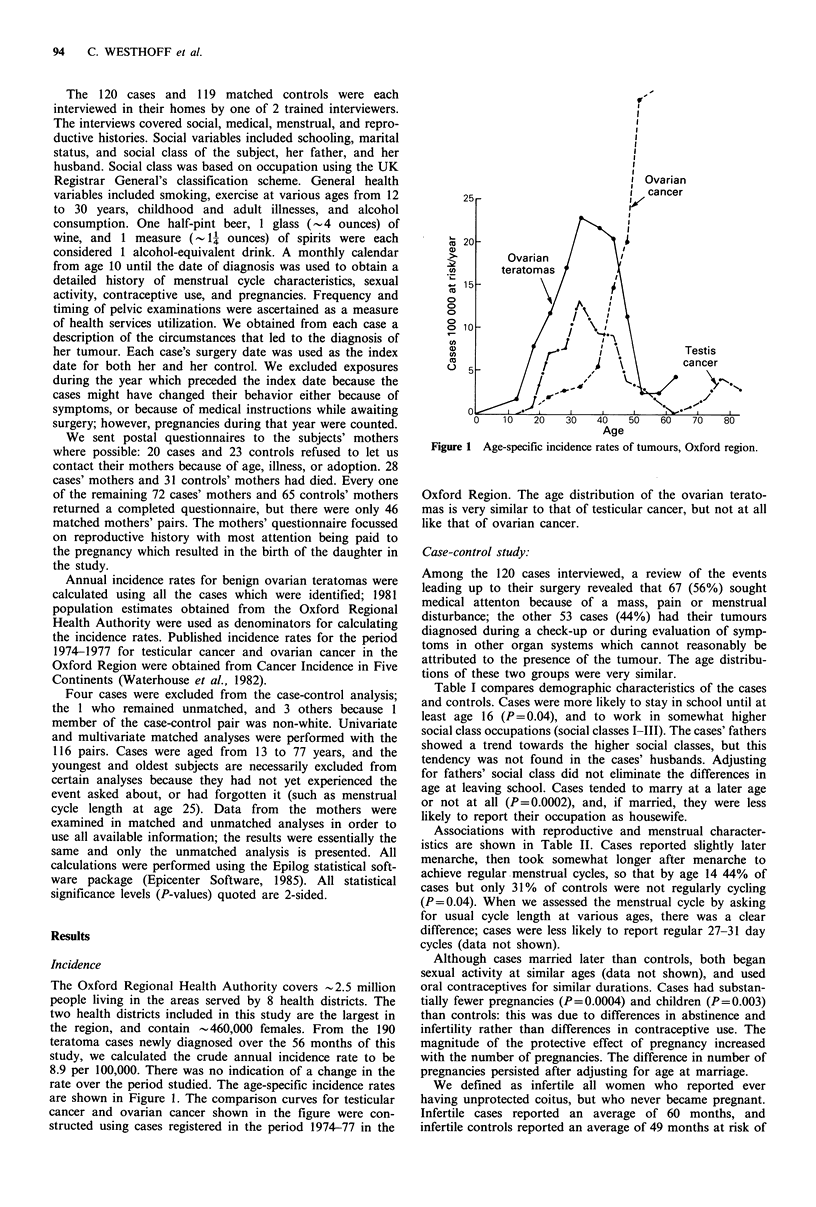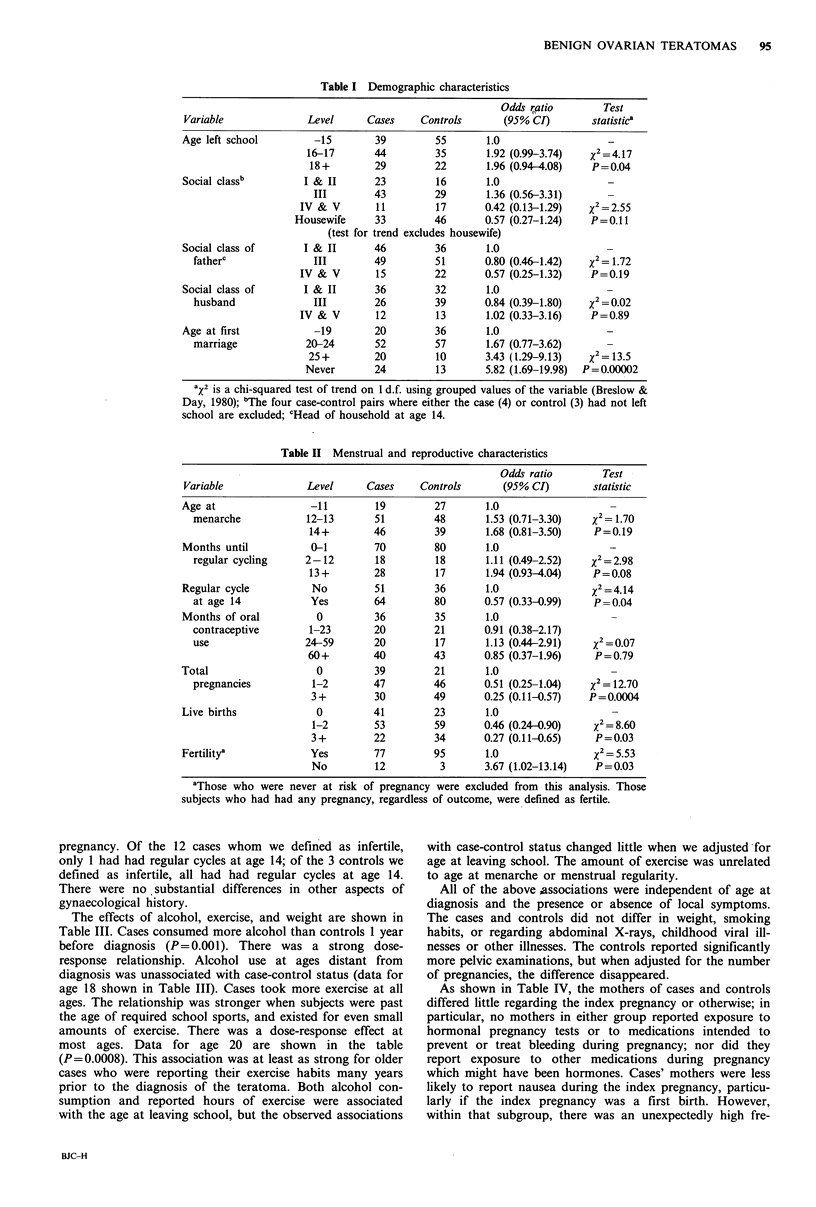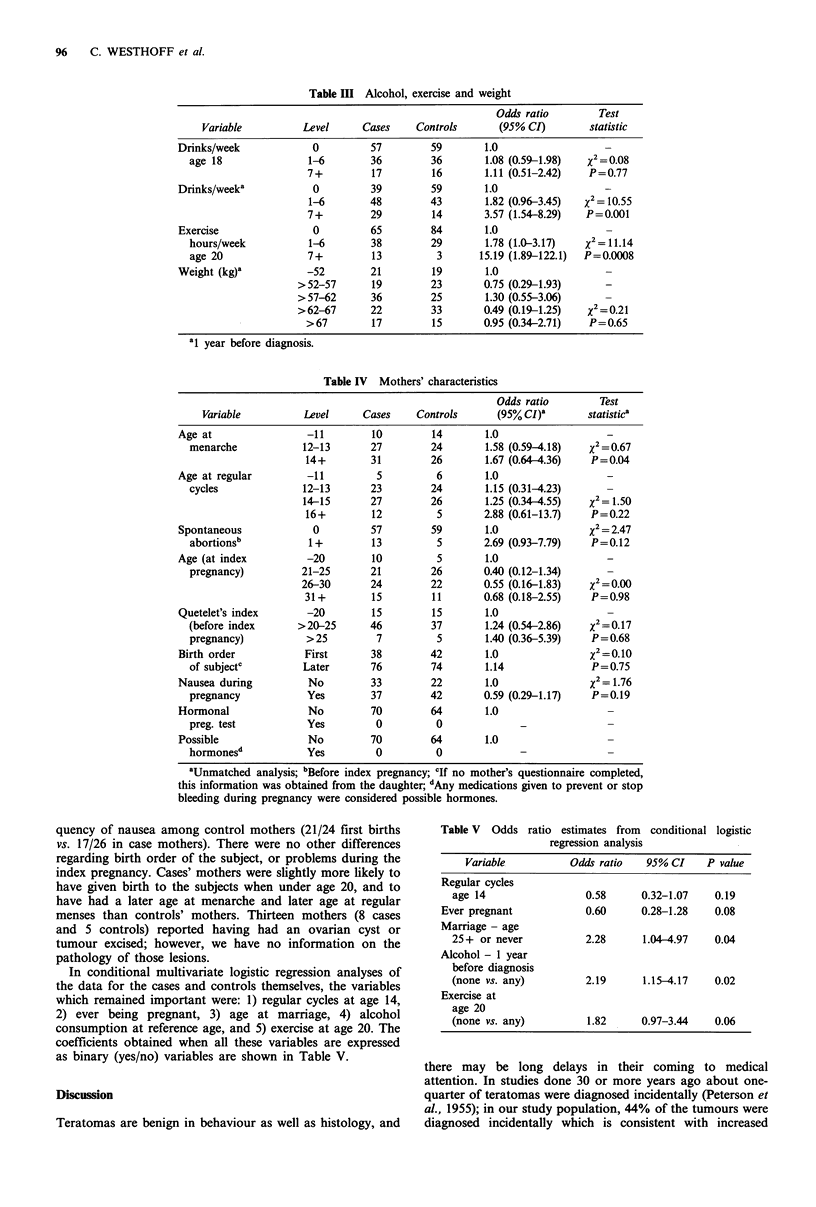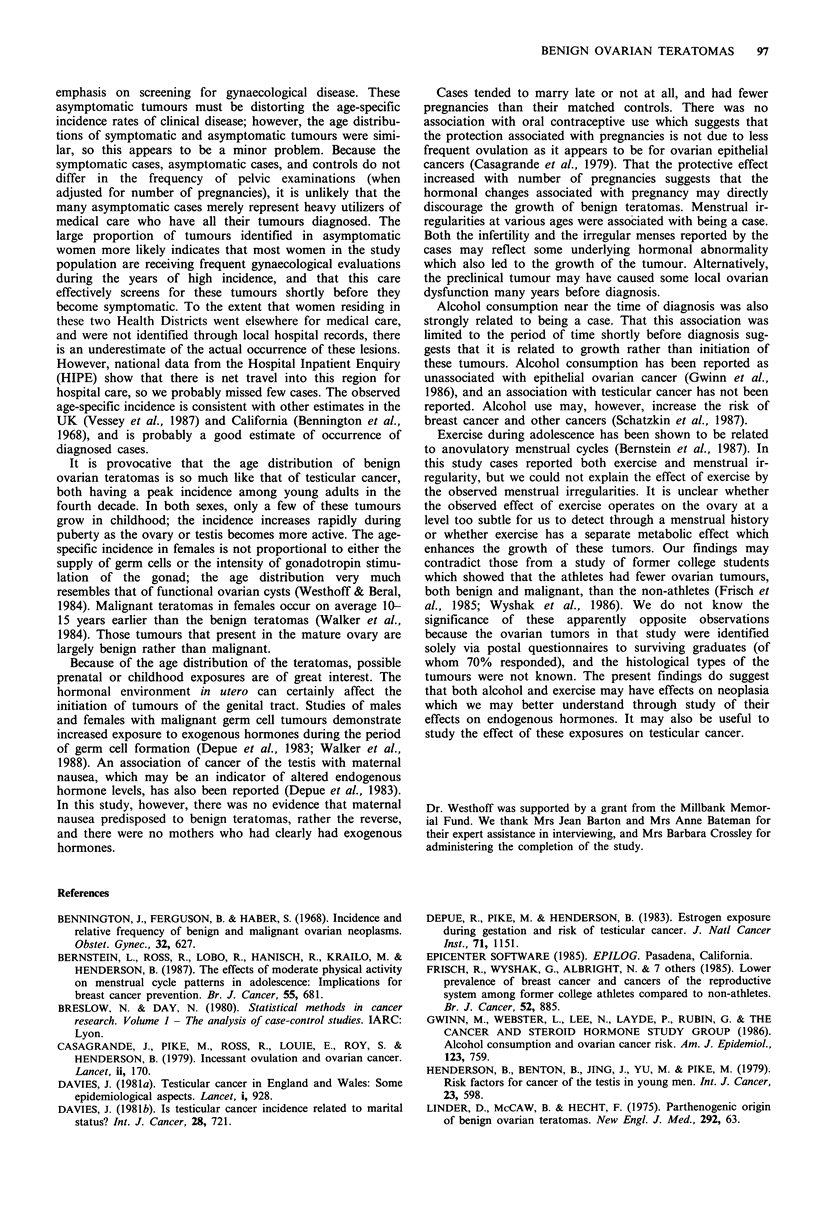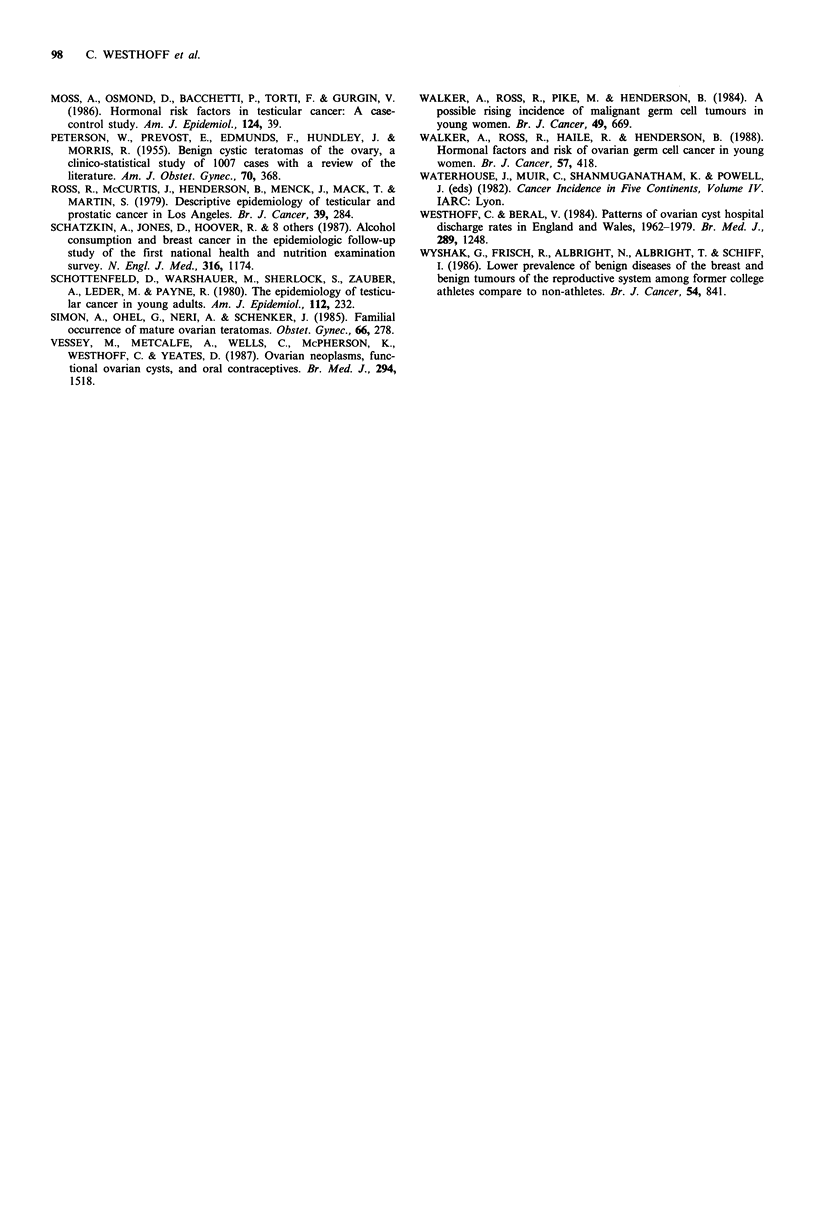# Benign ovarian teratomas: a population-based case-control study.

**DOI:** 10.1038/bjc.1988.171

**Published:** 1988-07

**Authors:** C. Westhoff, M. Pike, M. Vessey

**Affiliations:** Department of Community Medicine & General Practice, Radcliffe Infirmary, Oxford, UK.

## Abstract

We attempted to identify all cases of benign ovarian teratoma which occurred in two health districts in the UK during a 56 month period. The crude incidence was 8.9 cases/100,000 women. One hundred and twenty cases and 119 age-matched controls were interviewed to identify risk factors for this disease. In addition, 137 mothers completed postal questionnaires. Cases were older at leaving school, had higher social class occupations, were more often unmarried or married late, and had fewer children than controls. Oral contraceptive use was similar for both. Cases reported more exercise at all ages, and more alcohol consumption 1 year before diagnosis. Cases' mothers reported slightly less nausea during pregnancy than controls' mothers, and none of the mothers reported exogenous hormone exposure during the index pregnancy. In this study benign ovarian teratomas strongly resemble testicular cancer in their age distribution in the population. They also resemble testicular cancer in their association with educational status and marital status. There was, however, no similarity regarding prenatal hormone exposure. The increased risks associated with exercise and alcohol use were unexpected; we need further information about how these exposures affect the ovary, and whether they affect the testis.


*Br. J. Cancer* (1988), **58**, 93–98 © The Macmillan Press Ltd., 1988

### Benign ovarian teratomas: A population-based case-control study

C. Westhoff*, M. Pike† & M. Vessey

*Department of Community Medicine & General Practice and the Imperial Cancer Research Fund Epidemiology Unit, Radcliffe Infirmary, Oxford, UK.*

**Summary** We attempted to identify all cases of benign ovarian teratoma which occurred in two health districts in the UK during a 56 month period. The crude incidence was 8.9 cases/100,000 women. One hundred and twenty cases and 119 age-matched controls were interviewed to identify risk factors for this disease. In addition, 137 mothers completed postal questionnaires. Cases were older at leaving school, had higher social class occupations, were more often unmarried or married late, and had fewer children than controls. Oral contraceptive use was similar for both. Cases reported more exercise at all ages, and more alcohol consumption 1 year before diagnosis. Cases' mothers reported slightly less nausea during pregnancy than controls' mothers, and none of the mothers reported exogenous hormone exposure during the index pregancy. In this study benign ovarian teratomas strongly resemble testicular cancer in their age distribution in the population. They also resemble testicular cancer in their association with educational status and marital status. There was, however, no similarity regarding prenatal hormone exposure. The increased risks associated with exercise and alcohol use were unexpected; we need further information about how these exposures affect the ovary, and whether they affect the testis.

Teratoma means 'monstrous growth'. In the female, teratomas, sometimes called dermoid cysts, usually arise in the ovary, and are usually benign. Their monstrous character is quite apparent because the benign tumours contain tangled masses of hair mixed with teeth, cartilage, and bone. They are the most common ovarian neoplasm. In contrast, the malignant teratomas are very rare. Although the benign teratomas do sometimes cause symptoms, the usual reason for excising them is to ensure that there is no malignancy present. Little is known concerning the causes of these tumours, either benign or malignant. There are a few reports which suggest familial occurrence (Simon *et al.*, 1985). Genetic studies comparing tumour cells with somatic cells of the host have established that teratomas arise parthenogenetically from germ cells after the first meiotic division (Linder *et al.*, 1975). These cells (secondary oocytes) are found in the female ovary from the fourth month of prenatal life, and the total number is present by the eighth month of pregnancy.

One-quarter of all ovarian tumours arise from germ cells (teratomas, dysgerminomas); 98% of these are benign teratomas. Almost all testicular tumours are germ cell tumours (teratomas, seminomas), and in males it is exceedingly rare for these tumours to be benign. The incidence of testicular tumours has been increasing steadily for at least 50 years (Ross *et al.*, 1979; Davies, 1981*a*); a recent report suggests that malignant teratomas in females may also be increasing (Walker *et al.*, 1984).

The present study of benign ovarian teratomas was undertaken to learn about their incidence and distribution in a geographically defined population. We were particularly interested in whether they share the risk factors for testicular tumours. Several studies have identified an association between testicular cancer and maternal hormone use and maternal nausea during pregnancy (Henderson *et al.*, 1979; Schottenfeld *et al.*, 1980; Depue *et al.*, 1983), although 1 study has found no such association (Moss *et al.*, 1986). The only consistently reported risk factor for testicular cancer is cryptorchidism – a problem which has no obvious analogue in females. There is also evidence for an excess of testicular cancer among never-married and upper social class men (Ross *et al.*, 1979; Davies, 1981*b*), although this social class differential has not been identified among women with ovarian germ cell malignancies (Walker *et al.*, 1988).

### Materials and methods

In this geographically based case-control study, we attempted to identify through local hospital pathology records all women residing in the District Health Authority areas of Oxfordshire and West Berkshire who had had benign ovarian teratomas newly diagnosed between January 1, 1981 and August 31, 1985. There were 190 pathologically confirmed cases. Beginning in 1984, we attempted to locate these women and enrol them in the study.

The cases were traced through the family practitioner committees of the 2 health districts; 164 cases were located, 21 of whom had moved out of the area, and 2 of whom had died (1 of a contralateral malignant germ cell tumour, and one of multiple myeloma). The remaining 26 cases could not be readily traced. We sought to include in the study the 141 surviving cases located in the area. We did not contact any subject until we had permission from her gynaecologist and her general practitioner. General practitioners refused permission to contact 7 of these cases, usually because of family problems, and 14 cases declined to participate. In total, 120 cases were interviewed which was 85% (120/141) of those located alive in the 2 areas. Those who were not located or interviewed tended to be younger, to live in West Berkshire rather than Oxfordshire, and to have had their operations before the start of the study; we have no other information about them.

We attempted to match each case to a single control of the same age chosen from the list of the case's general practitioner. Using an alphabetical list of people registered with the practice, we aimed to select as the case's control the next woman on the list with at least one ovary whose birthday was within 6 months of the case's birthday. For 68 cases the first control selected was interviewed. For 52 cases the first control was not interviewed for the following reasons: 25 had moved or were untraced, one had died, the GP refused permission to contact 6, and 20 declined to participate. In these cases we selected the next woman of the same age from the list. Birthdate matching within 6 months was achieved for 116 pairs; 3 other controls were obtained by expanding the birthdate matching criterion to within 12 months. One case remained unmatched at the end of the study.

Present addresses:
*C. Westhoff, Department of Obstetrics and Gynecology, Columbia University College of Physicians & Surgeons, New York, USA.
†M. Pike, Department of Preventive Medicine, University of Southern California School of Medicine, Los Angeles, California, USA.
Correspondence: C. Westhoff at 630 West 168 St, New York, NY 10032.

The 120 cases and 119 matched controls were each interviewed in their homes by one of 2 trained interviewers. The interviews covered social, medical, menstrual, and reproductive histories. Social variables included schooling, marital status, and social class of the subject, her father, and her husband. Social class was based on occupation using the UK Registrar General's classification scheme. General health variables included smoking, exercise at various ages from 12 to 30 years, childhood and adult illnesses, and alcohol consumption. One half-pint beer, 1 glass (~4 ounces) of wine, and 1 measure (~1¼ ounces) of spirits were each considered 1 alcohol-equivalent drink. A monthly calendar from age 10 until the date of diagnosis was used to obtain a detailed history of menstrual cycle characteristics, sexual activity, contraceptive use, and pregnancies. Frequency and timing of pelvic examinations were ascertained as a measure of health services utilization. We obtained from each case a description of the circumstances that led to the diagnosis of her tumour. Each case's surgery date was used as the index date for both her and her control. We excluded exposures during the year which preceded the index date because the cases might have changed their behavior either because of symptoms, or because of medical instructions while awaiting surgery; however, pregnancies during that year were counted.

We sent postal questionnaires to the subjects' mothers where possible: 20 cases and 23 controls refused to let us contact their mothers because of age, illness, or adoption. 28 cases' mothers and 31 controls' mothers had died. Every one of the remaining 72 cases' mothers and 65 controls' mothers returned a completed questionnaire, but there were only 46 matched mothers' pairs. The mothers' questionnaire focussed on reproductive history with most attention being paid to the pregnancy which resulted in the birth of the daughter in the study.

Annual incidence rates for benign ovarian teratomas were calculated using all the cases which were identified; 1981 population estimates obtained from the Oxford Regional Health Authority were used as denominators for calculating the incidence rates. Published incidence rates for the period 1974–1977 for testicular cancer and ovarian cancer in the Oxford Region were obtained from Cancer Incidence in Five Continents (Waterhouse *et al.*, 1982).

Four cases were excluded from the case-control analysis; the 1 who remained unmatched, and 3 others because 1 member of the case-control pair was non-white. Univariate and multivariate matched analyses were performed with the 116 pairs. Cases were aged from 13 to 77 years, and the youngest and oldest subjects are necessarily excluded from certain analyses because they had not yet experienced the event asked about, or had forgotten it (such as menstrual cycle length at age 25). Data from the mothers were examined in matched and unmatched analyses in order to use all available information; the results were essentially the same and only the unmatched analysis is presented. All calculations were performed using the Epilog statistical software package (Epicenter Software, 1985). All statistical significance levels (*P*-values) quoted are 2-sided.

### Results

### *Incidence*

The Oxford Regional Health Authority covers ~2.5 million people living in the areas served by 8 health districts. The two health districts included in this study are the largest in the region, and contain ~460,000 females. From the 190 teratoma cases newly diagnosed over the 56 months of this study, we calculated the crude annual incidence rate to be 8.9 per 100,000. There was no indication of a change in the rate over the period studied. The age-specific incidence rates are shown in Figure 1. The comparison curves for testicular cancer and ovarian cancer shown in the figure were constructed using cases registered in the period 1974–77 in the Oxford Region. The age distribution of the ovarian teratomas is very similar to that of testicular cancer, but not at all like that of ovarian cancer.

**Figure 1** Age-specific incidence rates of tumours, Oxford region.

### *Case-control study:*

Among the 120 cases interviewed, a review of the events leading up to their surgery revealed that 67 (56%) sought medical attenton because of a mass, pain or menstrual disturbance; the other 53 cases (44%) had their tumours diagnosed during a check-up or during evaluation of symptoms in other organ systems which cannot reasonably be attributed to the presence of the tumour. The age distributions of these two groups were very similar.

Table I compares demographic characteristics of the cases and controls. Cases were more likely to stay in school until at least age 16 ($P = 0.04$), and to work in somewhat higher social class occupations (social classes I–III). The cases' fathers showed a trend towards the higher social classes, but this tendency was not found in the cases' husbands. Adjusting for fathers' social class did not eliminate the differences in age at leaving school. Cases tended to marry at a later age or not at all ($P = 0.0002$), and, if married, they were less likely to report their occupation as housewife.

Associations with reproductive and menstrual characteristics are shown in Table II. Cases reported slightly later menarche, then took somewhat longer after menarche to achieve regular menstrual cycles, so that by age 14 44% of cases but only 31% of controls were not regularly cycling ($P = 0.04$). When we assessed the menstrual cycle by asking for usual cycle length at various ages, there was a clear difference; cases were less likely to report regular 27–31 day cycles (data not shown).

Although cases married later than controls, both began sexual activity at similar ages (data not shown), and used oral contraceptives for similar durations. Cases had substantially fewer pregnancies ($P = 0.0004$) and children ($P = 0.003$) than controls: this was due to differences in abstinence and infertility rather than differences in contraceptive use. The magnitude of the protective effect of pregnancy increased with the number of pregnancies. The difference in number of pregnancies persisted after adjusting for age at marriage.

We defined as infertile all women who reported ever having unprotected coitus, but who never became pregnant. Infertile cases reported an average of 60 months, and infertile controls reported an average of 49 months at risk of

**Table I** Demographic characteristics

| *Variable* | *Level* | *Cases* | *Controls* | *Odds ratio (95% CI)* | *Test statistic*[a] |
|---|---|---|---|---|---|
| Age left school | –15 | 39 | 55 | 1.0 | – |
| | 16–17 | 44 | 35 | 1.92 (0.99–3.74) | $\chi^2=4.17$ |
| | 18+ | 29 | 22 | 1.96 (0.94–4.08) | $P=0.04$ |
| Social class[b] | I & II | 23 | 16 | 1.0 | – |
| | III | 43 | 29 | 1.36 (0.56–3.31) | – |
| | IV & V | 11 | 17 | 0.42 (0.13–1.29) | $\chi^2=2.55$ |
| | Housewife | 33 | 46 | 0.57 (0.27–1.24) | $P=0.11$ |
| | (test for trend excludes housewife) | | | | |
| Social class of father[c] | I & II | 46 | 36 | 1.0 | – |
| | III | 49 | 51 | 0.80 (0.46–1.42) | $\chi^2=1.72$ |
| | IV & V | 15 | 22 | 0.57 (0.25–1.32) | $P=0.19$ |
| Social class of husband | I & II | 36 | 32 | 1.0 | – |
| | III | 26 | 39 | 0.84 (0.39–1.80) | $\chi^2=0.02$ |
| | IV & V | 12 | 13 | 1.02 (0.33–3.16) | $P=0.89$ |
| Age at first marriage | –19 | 20 | 36 | 1.0 | – |
| | 20–24 | 52 | 57 | 1.67 (0.77–3.62) | – |
| | 25+ | 20 | 10 | 3.43 (1.29–9.13) | $\chi^2=13.5$ |
| | Never | 24 | 13 | 5.82 (1.69–19.98) | $P=0.00002$ |

[a]$\chi^2$ is a chi-squared test of trend on 1 d.f. using grouped values of the variable (Breslow & Day, 1980); [b]The four case-control pairs where either the case (4) or control (3) had not left school are excluded; [c]Head of household at age 14.

**Table II** Menstrual and reproductive characteristics

| *Variable* | *Level* | *Cases* | *Controls* | *Odds ratio (95% CI)* | *Test statistic* |
|---|---|---|---|---|---|
| Age at menarche | –11 | 19 | 27 | 1.0 | – |
| | 12–13 | 51 | 48 | 1.53 (0.71–3.30) | $\chi^2=1.70$ |
| | 14+ | 46 | 39 | 1.68 (0.81–3.50) | $P=0.19$ |
| Months until regular cycling | 0–1 | 70 | 80 | 1.0 | – |
| | 2–12 | 18 | 18 | 1.11 (0.49–2.52) | $\chi^2=2.98$ |
| | 13+ | 28 | 17 | 1.94 (0.93–4.04) | $P=0.08$ |
| Regular cycle at age 14 | No | 51 | 36 | 1.0 | $\chi^2=4.14$ |
| | Yes | 64 | 80 | 0.57 (0.33–0.99) | $P=0.04$ |
| Months of oral contraceptive use | 0 | 36 | 35 | 1.0 | – |
| | 1–23 | 20 | 21 | 0.91 (0.38–2.17) | |
| | 24–59 | 20 | 17 | 1.13 (0.44–2.91) | $\chi^2=0.07$ |
| | 60+ | 40 | 43 | 0.85 (0.37–1.96) | $P=0.79$ |
| Total pregnancies | 0 | 39 | 21 | 1.0 | – |
| | 1–2 | 47 | 46 | 0.51 (0.25–1.04) | $\chi^2=12.70$ |
| | 3+ | 30 | 49 | 0.25 (0.11–0.57) | $P=0.0004$ |
| Live births | 0 | 41 | 23 | 1.0 | – |
| | 1–2 | 53 | 59 | 0.46 (0.24–0.90) | $\chi^2=8.60$ |
| | 3+ | 22 | 34 | 0.27 (0.11–0.65) | $P=0.03$ |
| Fertility[a] | Yes | 77 | 95 | 1.0 | $\chi^2=5.53$ |
| | No | 12 | 3 | 3.67 (1.02–13.14) | $P=0.03$ |

[a]Those who were never at risk of pregnancy were excluded from this analysis. Those subjects who had had any pregnancy, regardless of outcome, were defined as fertile.

pregnancy. Of the 12 cases whom we defined as infertile, only 1 had had regular cycles at age 14; of the 3 controls we defined as infertile, all had had regular cycles at age 14. There were no substantial differences in other aspects of gynaecological history.

The effects of alcohol, exercise, and weight are shown in Table III. Cases consumed more alcohol than controls 1 year before diagnosis ($P=0.001$). There was a strong dose-response relationship. Alcohol use at ages distant from diagnosis was unassociated with case-control status (data for age 18 shown in Table III). Cases took more exercise at all ages. The relationship was stronger when subjects were past the age of required school sports, and existed for even small amounts of exercise. There was a dose-response effect at most ages. Data for age 20 are shown in the table ($P=0.0008$). This association was at least as strong for older cases who were reporting their exercise habits many years prior to the diagnosis of the teratoma. Both alcohol consumption and reported hours of exercise were associated with the age at leaving school, but the observed associations with case-control status changed little when we adjusted for age at leaving school. The amount of exercise was unrelated to age at menarche or menstrual regularity.

All of the above associations were independent of age at diagnosis and the presence or absence of local symptoms. The cases and controls did not differ in weight, smoking habits, or regarding abdominal X-rays, childhood viral illnesses or other illnesses. The controls reported significantly more pelvic examinations, but when adjusted for the number of pregnancies, the difference disappeared.

As shown in Table IV, the mothers of cases and controls differed little regarding the index pregnancy or otherwise; in particular, no mothers in either group reported exposure to hormonal pregnancy tests or to medications intended to prevent or treat bleeding during pregnancy; nor did they report exposure to other medications during pregnancy which might have been hormones. Cases' mothers were less likely to report nausea during the index pregnancy, particularly if the index pregnancy was a first birth. However, within that subgroup, there was an unexpectedly high fre-

**Table III** Alcohol, exercise and weight

| *Variable* | *Level* | *Cases* | *Controls* | *Odds ratio (95% CI)* | *Test statistic* |
|---|---|---|---|---|---|
| Drinks/week | 0 | 57 | 59 | 1.0 | – |
| age 18 | 1–6 | 36 | 36 | 1.08 (0.59–1.98) | $\chi^2=0.08$ |
| | 7+ | 17 | 16 | 1.11 (0.51–2.42) | $P=0.77$ |
| Drinks/week[a] | 0 | 39 | 59 | 1.0 | – |
| | 1–6 | 48 | 43 | 1.82 (0.96–3.45) | $\chi^2=10.55$ |
| | 7+ | 29 | 14 | 3.57 (1.54–8.29) | $P=0.001$ |
| Exercise | 0 | 65 | 84 | 1.0 | – |
| hours/week | 1–6 | 38 | 29 | 1.78 (1.0–3.17) | $\chi^2=11.14$ |
| age 20 | 7+ | 13 | 3 | 15.19 (1.89–122.1) | $P=0.0008$ |
| Weight (kg)[a] | -52 | 21 | 19 | 1.0 | – |
| | >52–57 | 19 | 23 | 0.75 (0.29–1.93) | – |
| | >57–62 | 36 | 25 | 1.30 (0.55–3.06) | – |
| | >62–67 | 22 | 33 | 0.49 (0.19–1.25) | $\chi^2=0.21$ |
| | >67 | 17 | 15 | 0.95 (0.34–2.71) | $P=0.65$ |

[a]1 year before diagnosis.

**Table IV** Mothers' characteristics

| *Variable* | *Level* | *Cases* | *Controls* | *Odds ratio (95% CI)*[a] | *Test statistic*[a] |
|---|---|---|---|---|---|
| Age at | -11 | 10 | 14 | 1.0 | – |
| menarche | 12–13 | 27 | 24 | 1.58 (0.59–4.18) | $\chi^2=0.67$ |
| | 14+ | 31 | 26 | 1.67 (0.64–4.36) | $P=0.04$ |
| Age at regular | -11 | 5 | 6 | 1.0 | – |
| cycles | 12–13 | 23 | 24 | 1.15 (0.31–4.23) | – |
| | 14–15 | 27 | 26 | 1.25 (0.34–4.55) | $\chi^2=1.50$ |
| | 16+ | 12 | 5 | 2.88 (0.61–13.7) | $P=0.22$ |
| Spontaneous | 0 | 57 | 59 | 1.0 | $\chi^2=2.47$ |
| abortions[b] | 1+ | 13 | 5 | 2.69 (0.93–7.79) | $P=0.12$ |
| Age (at index | -20 | 10 | 5 | 1.0 | – |
| pregnancy) | 21-25 | 21 | 26 | 0.40 (0.12–1.34) | – |
| | 26–30 | 24 | 22 | 0.55 (0.16–1.83) | $\chi^2=0.00$ |
| | 31+ | 15 | 11 | 0.68 (0.18–2.55) | $P=0.98$ |
| Quetelet's index | -20 | 15 | 15 | 1.0 | – |
| (before index | >20–25 | 46 | 37 | 1.24 (0.54–2.86) | $\chi^2=0.17$ |
| pregnancy) | >25 | 7 | 5 | 1.40 (0.36–5.39) | $P=0.68$ |
| Birth order | First | 38 | 42 | 1.0 | $\chi^2=0.10$ |
| of subject[c] | Later | 76 | 74 | 1.14 | $P=0.75$ |
| Nausea during | No | 33 | 22 | 1.0 | $\chi^2=1.76$ |
| pregnancy | Yes | 37 | 42 | 0.59 (0.29–1.17) | $P=0.19$ |
| Hormonal | No | 70 | 64 | 1.0 | – |
| preg. test | Yes | 0 | 0 | – | – |
| Possible | No | 70 | 64 | 1.0 | – |
| hormones[d] | Yes | 0 | 0 | – | – |

[a]Unmatched analysis; [b]Before index pregnancy; [c]If no mother's questionnaire completed, this information was obtained from the daughter; [d]Any medications given to prevent or stop bleeding during pregnancy were considered possible hormones.

quency of nausea among control mothers (21/24 first births *vs.* 17/26 in case mothers). There were no other differences regarding birth order of the subject, or problems during the index pregnancy. Cases' mothers were slightly more likely to have given birth to the subjects when under age 20, and to have had a later age at menarche and later age at regular menses than controls' mothers. Thirteen mothers (8 cases and 5 controls) reported having had an ovarian cyst or tumour excised; however, we have no information on the pathology of those lesions.

In conditional multivariate logistic regression analyses of the data for the cases and controls themselves, the variables which remained important were: 1) regular cycles at age 14, 2) ever being pregnant, 3) age at marriage, 4) alcohol consumption at reference age, and 5) exercise at age 20. The coefficients obtained when all these variables are expressed as binary (yes/no) variables are shown in Table V.

**Table V** Odds ratio estimates from conditional logistic regression analysis

| *Variable* | *Odds ratio* | *95% CI* | *P value* |
|---|---|---|---|
| Regular cycles age 14 | 0.58 | 0.32–1.07 | 0.19 |
| Ever pregnant | 0.60 | 0.28–1.28 | 0.08 |
| Marriage – age 25+ or never | 2.28 | 1.04–4.97 | 0.04 |
| Alcohol – 1 year before diagnosis (none *vs.* any) | 2.19 | 1.15–4.17 | 0.02 |
| Exercise at age 20 (none *vs.* any) | 1.82 | 0.97–3.44 | 0.06 |

### Discussion

Teratomas are benign in behaviour as well as histology, and there may be long delays in their coming to medical attention. In studies done 30 or more years ago about one-quarter of teratomas were diagnosed incidentally (Peterson *et al.*, 1955); in our study population, 44% of the tumours were diagnosed incidentally which is consistent with increased emphasis on screening for gynaecological disease. These asymptomatic tumours must be distorting the age-specific incidence rates of clinical disease; however, the age distributions of symptomatic and asymptomatic tumours were similar, so this appears to be a minor problem. Because the symptomatic cases, asymptomatic cases, and controls do not differ in the frequency of pelvic examinations (when adjusted for number of pregnancies), it is unlikely that the many asymptomatic cases merely represent heavy utilizers of medical care who have all their tumours diagnosed. The large proportion of tumours identified in asymptomatic women more likely indicates that most women in the study population are receiving frequent gynaecological evaluations during the years of high incidence, and that this care effectively screens for these tumours shortly before they become symptomatic. To the extent that women residing in these two Health Districts went elsewhere for medical care, and were not identified through local hospital records, there is an underestimate of the actual occurrence of these lesions. However, national data from the Hospital Inpatient Enquiry (HIPE) show that there is net travel into this region for hospital care, so we probably missed few cases. The observed age-specific incidence is consistent with other estimates in the UK (Vessey *et al.*, 1987) and California (Bennington *et al.*, 1968), and is probably a good estimate of occurrence of diagnosed cases.

It is provocative that the age distribution of benign ovarian teratomas is so much like that of testicular cancer, both having a peak incidence among young adults in the fourth decade. In both sexes, only a few of these tumours grow in childhood; the incidence increases rapidly during puberty as the ovary or testis becomes more active. The age-specific incidence in females is not proportional to either the supply of germ cells or the intensity of gonadotropin stimulation of the gonad; the age distribution very much resembles that of functional ovarian cysts (Westhoff & Beral, 1984). Malignant teratomas in females occur on average 10–15 years earlier than the benign teratomas (Walker *et al.*, 1984). Those tumours that present in the mature ovary are largely benign rather than malignant.

Because of the age distribution of the teratomas, possible prenatal or childhood exposures are of great interest. The hormonal environment *in utero* can certainly affect the initiation of tumours of the genital tract. Studies of males and females with malignant germ cell tumours demonstrate increased exposure to exogenous hormones during the period of germ cell formation (Depue *et al.*, 1983; Walker *et al.*, 1988). An association of cancer of the testis with maternal nausea, which may be an indicator of altered endogenous hormone levels, has also been reported (Depue *et al.*, 1983). In this study, however, there was no evidence that maternal nausea predisposed to benign teratomas, rather the reverse, and there were no mothers who had clearly had exogenous hormones.

Cases tended to marry late or not at all, and had fewer pregnancies than their matched controls. There was no association with oral contraceptive use which suggests that the protection associated with pregnancies is not due to less frequent ovulation as it appears to be for ovarian epithelial cancers (Casagrande *et al.*, 1979). That the protective effect increased with number of pregnancies suggests that the hormonal changes associated with pregnancy may directly discourage the growth of benign teratomas. Menstrual irregularities at various ages were associated with being a case. Both the infertility and the irregular menses reported by the cases may reflect some underlying hormonal abnormality which also led to the growth of the tumour. Alternatively, the preclinical tumour may have caused some local ovarian dysfunction many years before diagnosis.

Alcohol consumption near the time of diagnosis was also strongly related to being a case. That this association was limited to the period of time shortly before diagnosis suggests that it is related to growth rather than initiation of these tumours. Alcohol consumption has been reported as unassociated with epithelial ovarian cancer (Gwinn *et al.*, 1986), and an association with testicular cancer has not been reported. Alcohol use may, however, increase the risk of breast cancer and other cancers (Schatzkin *et al.*, 1987).

Exercise during adolescence has been shown to be related to anovulatory menstrual cycles (Bernstein *et al.*, 1987). In this study cases reported both exercise and menstrual irregularity, but we could not explain the effect of exercise by the observed menstrual irregularities. It is unclear whether the observed effect of exercise operates on the ovary at a level too subtle for us to detect through a menstrual history or whether exercise has a separate metabolic effect which enhances the growth of these tumors. Our findings may contradict those from a study of former college students which showed that the athletes had fewer ovarian tumours, both benign and malignant, than the non-athletes (Frisch *et al.*, 1985; Wyshak *et al.*, 1986). We do not know the significance of these apparently opposite observations because the ovarian tumors in that study were identified solely via postal questionnaires to surviving graduates (of whom 70% responded), and the histological types of the tumours were not known. The present findings do suggest that both alcohol and exercise may have effects on neoplasia which we may better understand through study of their effects on endogenous hormones. It may also be useful to study the effect of these exposures on testicular cancer.

Dr. Westhoff was supported by a grant from the Millbank Memorial Fund. We thank Mrs Jean Barton and Mrs Anne Bateman for their expert assistance in interviewing, and Mrs Barbara Crossley for administering the completion of the study.

### References

BENNINGTON, J., FERGUSON, B. & HABER, S. (1968). Incidence and relative frequency of benign and malignant ovarian neoplasms. *Obstet. Gynec.*, **32**, 627.

BERNSTEIN, L., ROSS, R., LOBO, R., HANISCH, R., KRAILO, M. & HENDERSON, B. (1987). The effects of moderate physical activity on menstrual cycle patterns in adolescence: Implications for breast cancer prevention. *Br. J. Cancer*, **55**, 681.

BRESLOW, N. & DAY, N. (1980). *Statistical methods in cancer research. Volume 1 – The analysis of case-control studies.* IARC: Lyon.

CASAGRANDE, J., PIKE, M., ROSS, R., LOUIE, E., ROY, S. & HENDERSON, B. (1979). Incessant ovulation and ovarian cancer. *Lancet*, **ii**, 170.

DAVIES, J. (1981*a*). Testicular cancer in England and Wales: Some epidemiological aspects. *Lancet*, **i**, 928.

DAVIES, J. (1981*b*). Is testicular cancer incidence related to marital status? *Int. J. Cancer*, **28**, 721.

DEPUE, R., PIKE, M. & HENDERSON, B. (1983). Estrogen exposure during gestation and risk of testicular cancer. *J. Natl Cancer Inst.*, **71**, 1151.

EPICENTER SOFTWARE (1985). *EPILOG*. Pasadena, California.

FRISCH, R., WYSHAK, G., ALBRIGHT, N. & 7 others (1985). Lower prevalence of breast cancer and cancers of the reproductive system among former college athletes compared to non-athletes. *Br. J. Cancer*, **52**, 885.

GWINN, M., WEBSTER, L., LEE, N., LAYDE, P., RUBIN, G. & THE CANCER AND STEROID HORMONE STUDY GROUP (1986). Alcohol consumption and ovarian cancer risk. *Am. J. Epidemiol.*, **123**, 759.

HENDERSON, B., BENTON, B., JING, J., YU, M. & PIKE, M. (1979). Risk factors for cancer of the testis in young men. *Int. J. Cancer*, **23**, 598.

LINDER, D., McCAW, B. & HECHT, F. (1975). Parthenogenic origin of benign ovarian teratomas. *New Engl. J. Med.*, **292**, 63.

MOSS, A., OSMOND, D., BACCHETTI, P., TORTI, F. & GURGIN, V. (1986). Hormonal risk factors in testicular cancer: A case-control study. *Am. J. Epidemiol.*, **124**, 39.

PETERSON, W., PREVOST, E., EDMUNDS, F., HUNDLEY, J. & MORRIS, R. (1955). Benign cystic teratomas of the ovary, a clinico-statistical study of 1007 cases with a review of the literature. *Am. J. Obstet. Gynec.*, **70**, 368.

ROSS, R., McCURTIS, J., HENDERSON, B., MENCK, J., MACK, T. & MARTIN, S. (1979). Descriptive epidemiology of testicular and prostatic cancer in Los Angeles. *Br. J. Cancer*, **39**, 284.

SCHATZKIN, A., JONES, D., HOOVER, R. & 8 others (1987). Alcohol consumption and breast cancer in the epidemiologic follow-up study of the first national health and nutrition examination survey. *N. Engl. J. Med.*, **316**, 1174.

SCHOTTENFELD, D., WARSHAUER, M., SHERLOCK, S., ZAUBER, A., LEDER, M. & PAYNE, R. (1980). The epidemiology of testicular cancer in young adults. *Am. J. Epidemiol.*, **112**, 232.

SIMON, A., OHEL, G., NERI, A. & SCHENKER, J. (1985). Familial occurrence of mature ovarian teratomas. *Obstet. Gynec.*, **66**, 278.

VESSEY, M., METCALFE, A., WELLS, C., McPHERSON, K., WESTHOFF, C. & YEATES, D. (1987). Ovarian neoplasms, functional ovarian cysts, and oral contraceptives. *Br. Med. J.*, **294**, 1518.

WALKER, A., ROSS, R., PIKE, M. & HENDERSON, B. (1984). A possible rising incidence of malignant germ cell tumours in young women. *Br. J. Cancer*, **49**, 669.

WALKER, A., ROSS, R., HAILE, R. & HENDERSON, B. (1988). Hormonal factors and risk of ovarian germ cell cancer in young women. *Br. J. Cancer*, **57**, 418.

WATERHOUSE, J., MUIR, C., SHANMUGANATHAM, K. & POWELL, J. (eds) (1982). *Cancer Incidence in Five Continents, Volume IV*. IARC: Lyon.

WESTHOFF, C. & BERAL, V. (1984). Patterns of ovarian cyst hospital discharge rates in England and Wales, 1962–1979. *Br. Med. J.*, **289**, 1248.

WYSHAK, G., FRISCH, R., ALBRIGHT, N., ALBRIGHT, T. & SCHIFF, I. (1986). Lower prevalence of benign diseases of the breast and benign tumours of the reproductive system among former college athletes compare to non-athletes. *Br. J. Cancer*, **54**, 841.